# Responsiveness of the German version of the Neck Disability Index in chronic neck pain patients: a prospective cohort study with a seven-week follow-up

**DOI:** 10.1186/s40945-022-00149-y

**Published:** 2022-10-17

**Authors:** Anke Langenfeld, Antonia Pia Gassner, Brigitte Wirth, Malin Beth Mühlemann, Luana Nyirö, Caroline Bastiaenen, Jaap Swanenburg

**Affiliations:** 1 Department of Chiropractic Medicine, Integrative Spinal Research (ISR Group), Balgrist University Hospital, University of Zurich, Forchstrasse 340, 8008 Zurich, Switzerland; 2 Care and Public Health Research Institute (CAPHRI), Maastricht University, Minderbroedersberg 4-6, 6211 LK Maastricht, the Netherlands; 3 School of Management and Law, Winterthur Institute of Health Economics, Zurich University of Applied Sciences, Gertrudstr. 15, 8400 Winterthur, Switzerland; 4 Department of Epidemiology, Maastricht University, Minderbroedersberg 4-6, 6211 LK Maastricht, the Netherlands; 5 University of Zurich, Raemistrasse 71, 8006 Zurich, Switzerland

**Keywords:** Chronic neck pain, German version of the Neck Disability Index, Responsiveness

## Abstract

**Background:**

The need for an efficient and feasible strategy to deal with neck pain has a high priority for many countries. Validated assessment tools like the Neck Disability Index (NDI) to evaluate the functional status of a neck pain patient are urgently needed to treat and to follow-up patients purposefully. A German version (NDI-G) was shown to be valid and reliable, but has so far not been tested for responsiveness. The aim of this study was to evaluate the NDI-G`s responsiveness.

**Methods:**

This was a prospective cohort study with a seven-week follow-up. Fifty chronic neck pain patients filled out NDI-G twice. Additionally, the Patients’ Global Impression of Change score (PGIC) was assessed at follow-up. Wilcoxon and Spearman tests were used to assess direction and strength of the association between the change in NDI-G and PGIC. The receiver operating characteristics method and the area under the curve (AUC) were calculated to assess sensitivity and specificity of the NDI-G change over time.

**Results:**

The Wilcoxon test showed statistically significant differences for NDI-G at baseline and follow-up in the total sample, the “clinically improved” and “clinically not improved” subgroups as indicated in the PGIC. Spearman test resulted in a moderate correlation between the NDI-G and the PGIC (r_S_ = -0.53, *p* = 0.01) at follow-up. AUC showed an acceptable discrimination [AUC = 0.78 (95% confidence interval 0.64 – 0.91)] of the NDI-G, with a cutoff score of 1.5, between clinically improved and clinically not improved patients, based on the PGIC.

**Conclusions:**

The NDI-G is responsive to change in chronic neck pain. Together with the results of a previous study on its validity and reliability, the NDI-G can be recommended for research and clinical settings in patients with neck pain in German speaking countries.

**Trial registration:**

NCT02676141. February 8, 2016.



## Background

Neck pain is one of the leading global causes of years lived with disability especially in the working population [1]. Its prevalence peaks around the age of 45 years [2]. The need for an efficient and feasible strategy to deal with neck pain problems has a high priority for many countries [2, 3]. The costs, either sick leave or treatment costs, have a huge economic impact on health care systems worldwide [4, 5]. This becomes even more significant with the ageing population, as the number of people living with sequelae of neck pain is increasing [3, 6]. Therefore, it is important to be able to assess a patient’s functional status properly and focus on his / her personal level of pain and dysfunction. In 1991, Howard Vernon developed the Neck Disability Index (NDI) [7, 8]. It is a widely used tool, which has been translated into many languages and has been tested for its reliability, validity and responsiveness in numerous studies [9]. Because translation can influence the methodological quality of a tool, it is important to test a tool for its psychometric properties after translation into other languages. The NDI has been translated into German (NDI-G) and validity and reliability have been shown [10]. Based on the international initiative of Consensus-based Standards for the selection of health Measurements Instruments (COSMIN), it is important to assess responsiveness to complete the evaluation of the NDI-G [11]. The aim of this study was to evaluate the responsiveness of the NDI-G in a German speaking population.

## Methods

### Study objective

The objective of this study was to evaluate the NDI-G`s sensitivity for change over time and the ability to distinguish between improved patients and non-improved patients, as assessed by the patients` impression of change.

### Study design, ethics, consent, and permissions

This study is a prospective cohort study with a seven-week follow-up. The study was performed in the Department of Chiropractic Medicine, Balgrist University Hospital, Zurich, Switzerland. The study was approved by the ethics committee of the canton of Zurich (BASEC 2015-00068) and registered at ClinicalTrials.gov (NCT0267614). All participants provided written informed consent.

### Participants

Participants were recruited from the Department of Chiropractic Medicine at Balgrist University Hospital and private practices. Inclusion criteria were chronic neck pain (neck pain > 12 weeks) [12], age > 18 years and ability to read, speak and write German. Exclusion criteria were the presence of any medical condition that contraindicates manual therapy applied to the cervical spine such as fractures, osteoporosis, vertebral arterial dysfunction, neoplasia in the cervical spine and infections. Additionally, no patients with systemic illnesses and cognitive impairments were included. During this time participants received common chiropractic care for their complaint such as spinal manipulation, trigger point therapy [13].

### Procedure

Diagnosis and medical history were assessed before filling out the questionnaires. NDI-G [10] (at baseline and after seven weeks) and, to assess possible improvement, the patient global impression of change (PGIC) [14, 15] (after seven weeks only) were sent to the participants via email using the REDCap electronic data capture tool hosted at the Balgrist University Hospital [16]. If a participant declined to provide the email address, paper versions were available.

### Assessments and outcome measures

The NDI is a short, paper–pencil self-reported questionnaire to assess disability in neck pain patients [8]. Originally it was developed for prognosis and reassessment of treatment [7]. The NDI consists of 10 items: pain intensity, personal care, lifting, reading, headaches, concentration, work, driving, sleeping and recreation. Each item can score up to five with a total score of 50. The lower the score, the less is self-rated disability [8]. Several studies investigated the responsiveness of the NDI in different languages e.g. Portuguese, Dutch, Norwegian, Japanese, patient cohorts and clinical settings [17–28]. The German version used in this study has been translated into German and tested for its validity and reliability in a previous study [10].

The PGIC scale is based on a seven-point Likert scale. It obtains patient’s report of improvement over time [14, 15]. The scale ranges from “much better”, “better”, “somewhat better”, “no change”, “somewhat worse”, “worse” to “much worse”. “Much better” is rated as 1 and “much worse” as 7 on the PGIC [14]. In this study, data was dichotomized: the PGIC ratings “much better”, “better” and somewhat better” (ratings 1–3) counted as “clinically improved”, the ratings from “no change” to “much worse” (ratings 4–7) counted as “clinically not improved” [19]. Additionally, the following general characteristics were collected from each patient at baseline: duration of chronic neck pain, onset of neck pain, age, gender, weight (kg), height (cm), medication and comorbidities.

### Statistical methods

Descriptive statistics were used to describe the characteristics of the patients in the total sample and the two sub-groups (clinically improved / clinically not improved) (Table 1). Missing values in the NDI-G were estimated as recommended by Vernon [8]. Questionnaires with more than three missing items were excluded from further analysis. Normality of data distribution was tested using the Shapiro–Wilk test. The raw change score was calculated as the difference between the NDI-G baseline scores and the follow-up scores [19]. The Wilcoxon test was used to compare changes in the NDI-G scores between baseline and follow-up of the “clinically improved” and “clinically not improved” group. Significance levels were set at *p* = 0.05. Spearman correlation was used to assess correlation between NDI-G change scores and PGIC. Coefficients were interpreted as excellent (> 0.9), good (0.7–0.9), moderate (0.5–0.69), fair (0.3–0.5), and little or none (0.0–0.3) [29]. To state the ability of detecting specificity and sensitivity for change over time and to estimate the minimal clinical important difference (MCID) the receiver operating characteristic method (ROC) (Youden Index) was used [30, 31]. Furthermore, the AUC was calculated. An AUC of < 0.70 indicates inadequate discrimination, between 0.70 and 0.80 indicates acceptable discrimination and > 0.80 indicates excellent discrimination [32]. SPSS Statistics 26 for Windows (Inc; Chicago, Illinois) was used for all statistical analyses.

## Results

**Fig. 1:**
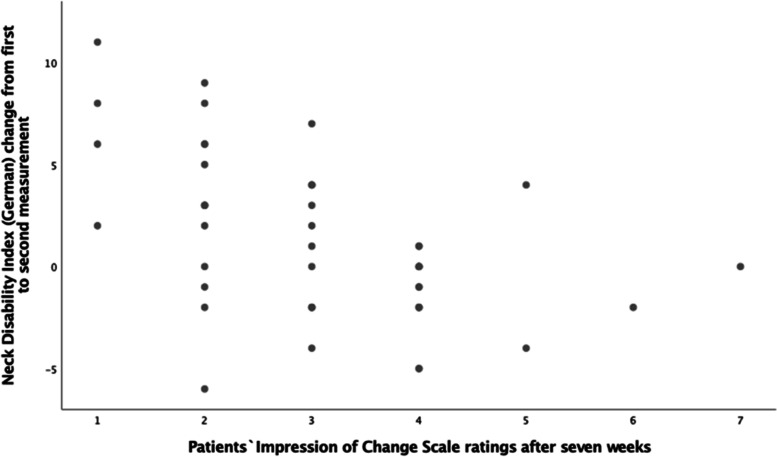
Scatterplot of NDI-G change score from first to second visit and patients` global impression of change scale (1 = much better, 2 = better, 3 = somewhat better, 4 = no change, 5 = somewhat worse, 6 = worse, 7 = much worse)

Fifty participants were recruited. Their mean age was 48.2 years (SD ± 15.1 years), 36 (72%) were female and 14 (28%) were male. Mean weight was 68.5 kg (SD ± 14.2 kg). Mean height was 171.7 cm (SD ± 9.4 cm). 50 patients completed baseline and 46 follow-up measurements. There were neither ceiling nor floor effects. All participants had chronic neck pain for more than 24 months except one patient who had neck pain for more than one year. 19 (38%) patients were complaining about frequent severe headaches, and 14 (28%) patients did not experience any headache. 39 (78%) patients did not take any pain medication, 11 (22%) took pain medication e.g., ibuprofen, paracetamol and triptan on a frequent basis. 31 (62%) had no other comorbidities, 19 (38%) had additional problems e.g., temporomandibular disorder, shoulder impingement syndrome, and cardiovascular impairments. At baseline, four participants did not answer one item (three: driving, one: reading). Two patients gave the reason of driving no car, two gave no reasons. At follow-up, two participants did not answer one question (driving), but did not give any reasons. All data sets were kept in the final analysis. Dichotomization according to PGIC resulted in 17 (37%) “clinically not improved” and 29 (63%) “clinically improved” participants (Table 1, Fig. 1).

**Table 1:** Demographics (mean and standard deviation (SD) at baseline and NDI-G scores at baseline, at seven weeks follow-up and change scores between both measurement points, for the total sample and split up for patients` global impression of change as clinically improved (ratings 1–3) and clinically not improved (rating 4–7) patients

	Total sample (*n* = 50)	Clinically improved (*n* = 29)	Clinically not improved (*n* = 17)
Sex	14 male / 36 female	7 male / 22 female	7 male / 14 female
Age (y)	48.2 (15.1)	46.1 (14.3)	51.1 (15.9)
Weight (kg)	68.5 (14.2)	68.1 (14.8)	69.0 (13.6)
Height (cm)	171.7 (9.4)	171.5 (9.6)	171.9 (9.5)
NDI-G baseline [mean (SD)]	12.0 (6.2)	11.4 (5.6)	12.8 (6.9)
NDI-G follow-up [mean (SD)]	10.6 (5.9)	8.7 (5.1)	13.8 (5.9)
NDI-G change score [mean (SD)]	1.3 (3.9)	2.6 (4.0)	-1.0 (2.2)

**Fig. 2:**
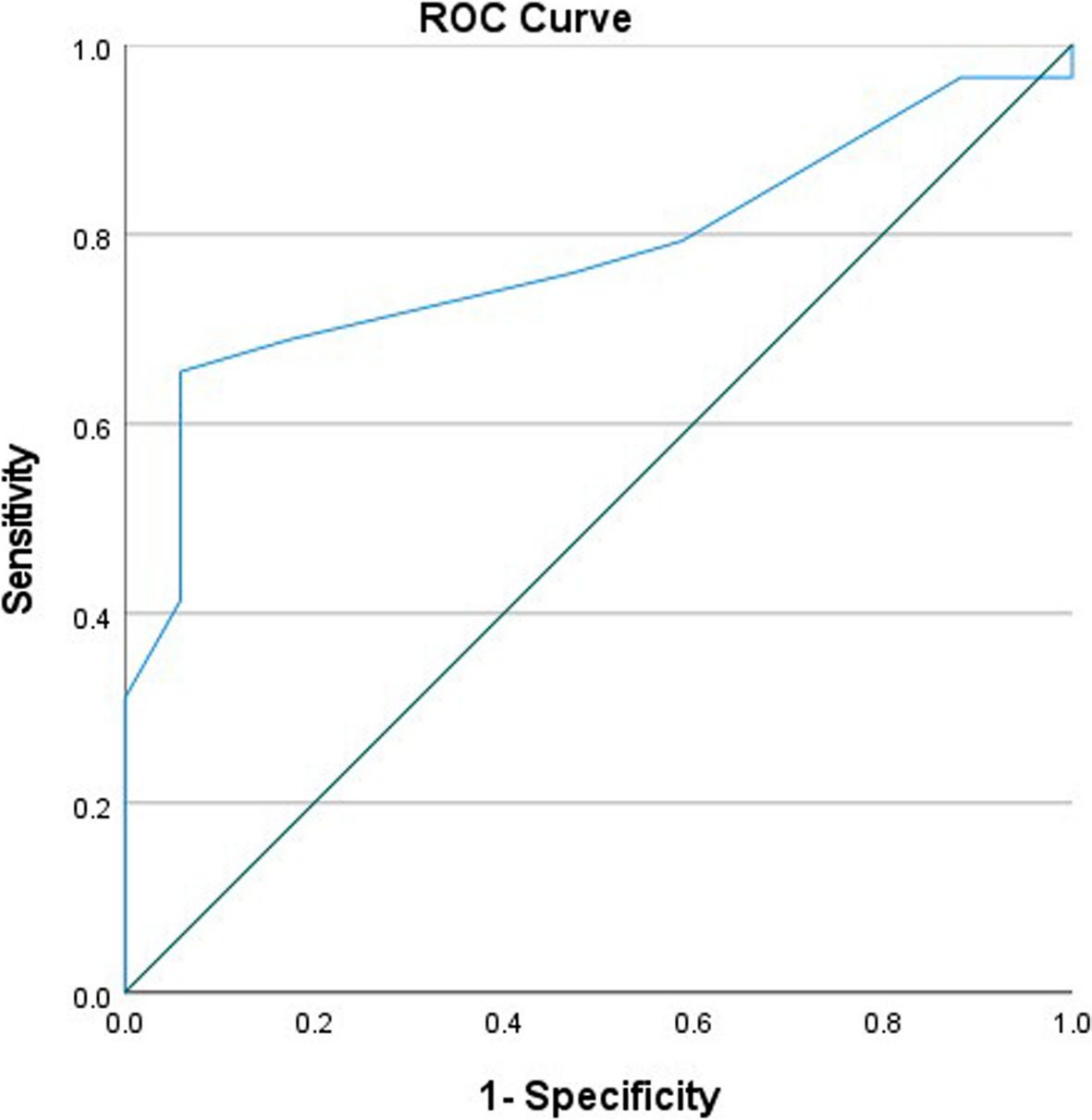
Receiver operating characteristics curve (ROC) of the NDI-G change scores, “clinically improved” and “clinically not improved” patients as indicated in the patient`s global impression of change scale. The area under the curve (AUC) is 0.78

Mean change score in NDI-G was 1.30 (SD 3.9). The result of the Wilcoxon test stated a significant difference between the baseline and the follow-up NDI-G total scores in the total sample (*p* = 0.04), the “clinically improved” sample (*p* < 0.001) and the “clinically not improved” sample (*p* = 0.05). There was a significant moderate negative correlation between NDI-G change scores and the total sample PGIC ratings (r_S_ = -0.506, *p* < 0.001). The ROC curve showed an AUC of 0.78 (95% confidence interval 0.64 – 0.91) (Fig. 2). The cutoff score was 1.5 (sensitivity = 0.655, specificity = 0.941). This indicates an acceptable discrimination of the NDI-G change score of 2, between patients with a clinical improvement and those who did not report improvement.

## Discussion

The aim of this study was to evaluate the NDI-G`s sensitivity to change over time and its ability to distinguish between chronic neck pain patients categorized as “clinically improved” and “clinically not improved”, as assessed by the PGIC. NDI-G and PGIC correlated moderately at seven weeks follow-up. The ROC using the PGIC as an external anchor resulted in an AUC value of 0.78 (95% confidence interval 0.64 – 0.91), which indicates moderate, but acceptable responsiveness [33].

Several studies investigated the responsiveness of the NDI in different languages, patient cohorts and clinical settings [17–28]. Six studies evaluated responsiveness in chronic neck pain patients [17, 19, 21, 24, 26, 28]. The correlation coefficients between NDI change scores, different external anchors ranged from 0.32 for the PGIC (Pereira, Cruz, 2015) and 0.71 global perceived effect (GPE) [24].

One important aspect that could have influenced the outcome in such responsiveness studies is the measurement tool which was used as an external anchor. Tools used were the functional rating index (FRI) [26], global perceived effect (GPE) [24], global rating of change (GRS) [17, 21] and patients` global impression of change scale (PGIC) [19]. Eventually, this might influence comparison of the results as the different measurement tools might have different underlying constructs. AUCs ranged between 0.33 [26] and 0.96 [24]. Nevertheless, the present study`s AUC results are nicely in line with the results of Young et al., who reported an AUC of 0.79 [17]. Interestingly, they used a comparable short follow-up time of three weeks and included participants with or without upper extremity symptoms [17]. Furthermore, the results of the present study are close to the results of Johansen et al., 2014, who reported a AUC of 0.70 [21], but contrary to Young et al., 2009, Johansen et al. used a follow-up of two years [21]. Compared to both studies, the sample size of the present study, with a follow-up sample of 46, was rather small, but reached comparable results. All other studies found deviant results. Monticone and colleagues, 2015 reported an AUC of 0.96 for the Italian version [24], whereas Salehi et al., 2019 reported an AUC of 0.33 for the Persian version [26] and Pereira et al., 2015, with a similar follow up and external anchor (PGIC) as the present study, reported and AUC of 0.59 for the Portuguese Version [19]. Another study be Takeshita et al., 2013 that used the PGIC as external anchor, did not report any AUC [28]. Additionally, the patients in these studies [19, 28] differed in baseline characteristics from those in the present study the mean value of the NDI was higher and the patients were treated by multimodal physiotherapy and surgery [19, 28].

The main limitation of the present study was its sample size of 50 patients at baseline, which is the minimal sample size recommended [34]. Nevertheless, the results are well comparable to those of NDI versions in other languages, which might indicate robustness of the results despite the limited sample size. Additionally, the chiropractic treatment was not standardized. However, outcome measures were not compared between, but only within individuals and thus, treatment characteristics might have affected recovery, but not the study results. Furthermore, generalizability of the results is limited, due to the clinical characteristics of the sample, e.g. low baseline NDI scores.

## Conclusion

NDI-G emerged from this study as sensitive to capture changes over time. Its responsiveness is acceptable and comparable to similar studies on the NDI in other languages. Together with the results of the previous study on the reliability and validity of the NDI-G, NDI-G can be recommended for research and clinical settings in neck pain in German speaking countries.

## Data Availability

The datasets used and/or analysed during the current study are available from the corresponding author on reasonable request.

## Abbreviations

AUC: Area Under the Curve
CRF: Clinical Report Form
MCID: Minimal Clinical Important Difference
NDI: Neck Disability Index
NDI-G: German Version of the Neck Disability Index
PGIC: Patients’ Global Impression of Change
ROC: Receiver Operating Characteristic
SD: Standard Deviation
y: Years

## References

[1] Vos T, Lim SS, Abbafati C, Abbas KM, Abbasi M, Abbasifard M (2020). Global burden of 369 diseases and injuries in 204 countries and territories, 1990–2019: a systematic analysis for the Global Burden of Disease Study 2019. The Lancet.

[2] Hoy D, March L, Brooks P, Blyth F, Woolf A, Bain C (2014). The global burden of low back pain: estimates from the Global Burden of Disease 2010 study. Ann Rheum Dis.

[3] Vos T, Allen C, Arora M, Barber RM, Bhutta ZA, Brown A (2016). Global, regional, and national incidence, prevalence, and years lived with disability for 310 diseases and injuries, 1990–2015: a systematic analysis for the Global Burden of Disease Study 2015. Lancet.

[4] Kleinman N, Patel AA, Benson C, Macario A, Kim M, Biondi DM (2014). Economic burden of back and neck pain: effect of a neuropathic component. Popul Health Manag.

[5] Bernfort L, Gerdle B, Rahmqvist M, Husberg M, Levin LA (2015). Severity of chronic pain in an elderly population in Sweden–impact on costs and quality of life. Pain.

[6] Carone G, Costello D, Diez Guardia N, Mourre G, Przywara B, Salomaki A. The economic impact of ageing populations in the EU25 Member States. European Communities, Directorate-General for Economic and Financial Affairs; 2005.

[7] Vernon H, Mior S (1991). The Neck Disability Index: a study of reliability and validity. J Manipulative Physiol Ther.

[8] Vernon H (2008). The Neck Disability Index: state-of-the-art, 1991–2008. J Manipulative Physiol Ther.

[9] Bobos P, MacDermid JC, Walton DM, Gross A, Santaguida PL (2018). Patient-reported outcome measures used for neck disorders: an overview of systematic reviews. J Orthop Sports Phys Ther.

[10] Swanenburg J, Humphreys K, Langenfeld A, Brunner F, Wirth B (2014). Validity and reliability of a German version of the Neck Disability Index (NDI-G). Man Ther.

[11] Mokkink LB, Terwee CB, Patrick DL, Alonso J, Stratford PW, Knol DL (2010). The COSMIN checklist for assessing the methodological quality of studies on measurement properties of health status measurement instruments: an international Delphi study. Qual Life Res.

[12] Gross A, Miller J, D’Sylva J, Burnie SJ, Goldsmith CH, Graham N (2010). Manipulation or mobilisation for neck pain: a Cochrane Review. Man Ther.

[13] Humphreys BK, Peterson CK, Muehlemann D, Haueter P (2010). Are Swiss chiropractors different than other chiropractors? Results of the job analysis survey 2009. J Manipulative Physiol Ther.

[14] Farrar JT, Young JPJ, LaMoreaux L, Werth JL, Poole RM (2001). Clinical importance of changes in chronic pain intensity measured on an 11-point numerical pain rating scale. Pain.

[15] Swanenburg J, Gruber C, Brunner F, Wirth B (2015). Patients' and therapists' perception of change following physiotherapy in an orthopedic hospital's outpatient clinic. Physiother Theory Pract.

[16] Harris PA. Research electronic data capture (REDCap) - A metadata-driven methodology and workflow process for providing translational research informatics support. In: Taylor R, Thielke R, Payne J, Gonzalez N, Gonde JG, editors. Elsevier, Journal of Biomedical Informatics. 2009. p. 377–81. 10.1016/j.jbi.2008.08.010 PMC2700030 18929686

[17] Young BA, Walker MJ, Strunce JB, Boyles RE, Whitman JM, Childs JD (2009). Responsiveness of the Neck Disability Index in patients with mechanical neck disorders. Spine J.

[18] Young IA, Dunning J, Butts R, Cleland JA, Fernandez-de-Las-Penas C (2018). Psychometric properties of the Numeric Pain Rating Scale and Neck Disability Index in patients with cervicogenic headache. Cephalalgia.

[19] Pereira M, Cruz EB, Domingues L, Duarte S, Carnide F, Fernandes R (2015). Responsiveness and Interpretability of the Portuguese Version of the Neck Disability Index in patients with chronic neck pain undergoing physiotherapy. Spine (Phila Pa 1976).

[20] Vos CJ, Verhagen AP, Koes BW (2006). Reliability and responsiveness of the Dutch version of the Neck Disability Index in patients with acute neck pain in general practice. Eur Spine J.

[21] Johansen JB, Roe C, Bakke E, Mengshoel AM, Andelic N (2014). Reliability and responsiveness of the Norwegian version of the Neck Disability Index. Scand J Pain.

[22] Chien A, Lai DM, Cheng CH, Wang SF, Hsu WL, Wang JL (2015). Responsiveness of the Chinese versions of the Japanese Orthopaedic Association Cervical Myelopathy Evaluation Questionnaire and Neck Disability Index in postoperative patients with cervical spondylotic myelopathy. Spine (Phila Pa 1976).

[23] Ailliet L, Rubinstein SM, de Vet HC, van Tulder MW, Terwee CB (2015). Reliability, responsiveness and interpretability of the neck disability index-Dutch version in primary care. Eur Spine J.

[24] Monticone M, Ambrosini E, Vernon H, Brunati R, Rocca B, Foti C (2015). Responsiveness and minimal important changes for the Neck Disability Index and the Neck Pain Disability Scale in Italian subjects with chronic neck pain. Eur Spine J.

[25] Stefanovitch-Lawbuary N, Amirfeyz R, Lovell R, Bannister G (2019). Reliability and responsiveness of patient-reported outcome measures of neck disability to physical therapy: Comparison of the Copenhagen, Northwick Park, and Neck Bournemouth Questionnaires and the Neck Disability Index. J Manipulative Physiol Ther.

[26] Salehi R, Negahban H, Saghayezhian N, Saadat M (2019). The responsiveness of the Persian Version of Neck Disability Index and functional rating index following physiotherapy intervention in people with chronic neck pain. Iran J Med Sci.

[27] Young I, Dunning J, Butts R, Mourad F, Cleland J (2019). Reliability, construct validity, and responsiveness of the neck disability index and numeric pain rating scale in patients with mechanical neck pain without upper extremity symptoms. Physiother Theory Pract.

[28] Takeshita K, Hosono N, Kawaguchi Y, Hasegawa K, Isomura T, Oshima Y (2013). Validity, reliability and responsiveness of the Japanese version of the Neck Disability Index. J Orthop Sci.

[29] Landis JR, Koch GG (1977). The measurement of observer agreement for categorical data. Bioemetrics.

[30] Youden WJ (1950). Index for rating diagnostic tests. Cancer.

[31] Schisterman EF, Faraggi D, Reiser B, Hu J (2008). Youden Index and the optimal threshold for markers with mass at zero. Stat Med.

[32] Hosmer DW, Lemenshow S (2005). Applied logistic regression.

[33] De Vet HC, Terwee CB, Mokkink LB, Knol DL (2011). Measurement in medicine.

[34] Mokkink LiB, Prinsen CA, Donald LP, Alonso J, Bouter LM, de Vet HC, et al. Cosmin Study Design checklist for Patient-reported outcome measurment instruments [Online pdf]. COSMIN: COSMIN; 2019 [updated 2019.

